# The ACTyourCHANGE study protocol: promoting a healthy lifestyle in patients with obesity with Acceptance and Commitment Therapy—a randomized controlled trial

**DOI:** 10.1186/s13063-021-05191-y

**Published:** 2021-04-20

**Authors:** Anna Guerrini Usubini, Roberto Cattivelli, Emanuele Maria Giusti, Francesco Vailati Riboni, Giorgia Varallo, Giada Pietrabissa, Gian Mauro Manzoni, Simone Consoli, Ilaria Bastoni, Valentina Granese, Clarissa Volpi, Valentina Villa, Annalisa Caretti, Michela Bottacchi, Gianluca Castelnuovo, Enrico Molinari

**Affiliations:** 1 Istituto Auxologico Italiano IRCCS, Psychology Research Laboratory, Milan, Italy; 2 Department of Psychology, Catholic University of Milan, Milan, Italy; 3 Department of Psychology, eCampus University, Novedrate, Italy

**Keywords:** Acceptance and Commitment Therapy (ACT), Psychological flexibility, Cognitive Behavioral Therapy (CBT), Healthy lifestyle, Obesity rehabilitation

## Abstract

**Background:**

As treatment of choice in promoting psychological flexibility, Acceptance and Commitment Therapy (ACT) was found to be effective in several conditions, and among different populations, including weight management in individuals with obesity. However, the mechanism of action of psychological flexibility is less known. The aim of the present study is, within the context of a brief ACT intervention for behavioral change and behavioral maintenance of a healthy lifestyle in a sample of inpatients with obesity, to explore the effect of each subcomponent of the psychological flexibility model on treatment processes and outcomes.

**Methods:**

A randomized controlled trial will be conducted. Ninety Italian adult inpatients with obesity attending a rehabilitation program for weight loss will be randomly allocated into three experimental conditions targeting respectively each subcomponent of the psychological flexibility model: group Engage focused on values-oriented behaviors, group Openness focused on acceptance and cognitive defusion, and group Awareness focused on being present and aware of thoughts, feelings, and behaviors at every moment. Weight, BMI (kg/m^2^), the Psychological General Well-Being Inventory (PGWBI), the Outcome Questionnaire-45.2 (OQ-45.2), the Depression Anxiety and Stress Scale (DASS-21), the Difficulties in Emotion Regulation Scale (DERS), the Dutch Eating Behaviors Questionnaire (DEBQ), the Brief Values Inventory (BVI), the Committed Action Questionnaire (CAQ), the Italian-Cognitive Fusion Questionnaire (I-CFQ), the Five Facet Mindfulness Questionnaire (FFMQ), and the Acceptance and Action Questionnaire (AAQ-II) will be assessed at the beginning (time 0), at the end of psychological intervention (time 1), and after 3 (time 2) and 6 months (time 3) and 9 months (time 4) from discharge. During the following month after discharge, outpatients will be monitored in their adherence to a healthy lifestyle, using a wearable device.

To assess the effectiveness of the intervention, mixed between-within 3 (conditions) × 4 (times) repeated measure ANOVAs will be conducted to examine changes from time 0 to time 1, 2, 3, and 4 in means of weight, BMI, and means of scores PGWBI, OQ-45.2, DASS, DERS, DEBQ, AAQ-II, BVI, CAQ, I-CFQ, and FFMQ, between three groups.

**Discussion:**

This study will contribute to clarify the mechanism of action of each subcomponent of the psychological flexibility model and understand its impact on the promotion of a healthy lifestyle.

**Trial registration:**

ClinicalTrials.govNCT04474509. Registered on July 4, 2020

## Background

Obesity is one of the most serious health problems in global public health [1]. Recent estimates pointed out that over the last years obesity reached epidemic proportions, and its prevalence is still rising. In 2016, over 1.9 billion adults in the world were overweight and, of these, more than 650 million were obese [2].

Defined as an excess of body weight, obesity is a significant risk factor for a plethora of physical, psychological, and social problems, all of which can heavily impact health, quality of life, and global functioning. Obesity is frequently associated with many physical comorbidities, including type II diabetes mellitus, cardiovascular disease, hypertension, kidney failure, and osteoarthritis [3]; psychological problems such as depression, feelings of shame, low self-esteem, and stigma [4]; and eating disorders, as well as social and economic impairment [5].

Given the complex nature of the phenomenon, comprehensive multidisciplinary and multi-component lifestyle interventions for the management of obesity in adults are recommended. They include nutrition and dieting, physical activity, and psychological support, aimed at fostering the adoption of a healthy lifestyle through the Cognitive Behavioral Therapy-based interventions considered the gold standard for the treatment of obesity [6].

Even if such programs have been recognized as effective in promoting healthy lifestyle adoption, the maintenance of behavioral change remains challenging. Most obese or overweight people who attend a weight loss rehabilitation program fail to maintain a healthy lifestyle over time. As a consequence, they regain about one-third of the weight lost over the following year after treatment [7, 8]. This evidence has spurred research to investigate which factors represent barriers to weight loss maintenance and which factors may influence the adoption of healthy lifestyle habits.

Forman and Butryn’s conceptual model [9] suggest that long-standing adherence to a healthy lifestyle is partially due to some self-regulation skills, such as distress tolerance, values clarity, metacognitive awareness, and behavioral commitment. Such skills were found to play a protective role against the excessive responsiveness to such internal (such as emotions) and external (the availability of tasty food in the modern environment) cues that motivate people to eat palatable food in response to negative internal states, as in case of emotional eating.

Emotional eating refers to eating in response to unpleasant emotional states [10]. Since emotional eating has been associated with elevated consumption of high-calorie and high fat, it does not surprise that emotional eating was found to be strongly related to obesity [11] both in adults and in the younger population [12, 13].

The link between negative emotions and emotional eating has been well-established both in general [14] and in obese population [15]. Many studies suggested that dysfunctional eating habits, such as emotional eating, may emerge as a failure of emotion regulation strategies that allow individuals to regulate their own emotions by modulating their responses [16, 17]. As a result, individuals eat in an attempt to regulate their negative internal states.

The treatment of choice to promote self-regulation skills is Acceptance and Commitment Therapy (ACT [18];). ACT is one of the recent third-wave Cognitive Behavioral Therapies (CBTs) rose in the last 20 years. It is a transdiagnostic approach aimed at promoting psychological flexibility defined as the ability of “being in contact with the present moment fully as a conscious human being and, basing on what the situation affords, changing or persisting in behavior in the service of chosen values” [18]. The promotion of psychological flexibility is based on three subcomponents of the psychological flexibility model: openness, awareness, and engagement. Openness refers to the willingness to develop an open and acceptable attitude toward one’s personal internal states such as thoughts, emotions, and bodily sensations; awareness refers to the ability to act intentionally with awareness about personal thought and sensations, without automatically reacting; engagement refers to engage oneself in committed behaviors related to personal values, that is chosen life directions [19].

Over the years, ACT has been successfully applied in various pathological conditions, including obesity management both in adult and young populations [20–22]. In an RCT [23] comparing a standard CBT-based group psychological intervention with an ACT-based group psychological intervention within an in-hospital multidisciplinary rehabilitation program for weight loss for patients with obesity, it was found that, since there were no significant differences between groups from pre- to post-interventions in weight reduction (both groups showed a decrease of weight and BMI from pre- to post-intervention), only participants in the ACT condition were able to maintain weight lost during the intervention, over the following 6 months after discharge. About the psychological conditions, CBT was greater than ACT in producing improvement in the psychological conditions (subjective well-being, psychological problems, life functioning, risk for self-harm or harm others) from pre- to post-intervention. On the contrary, at 6-month follow-up, the effect of the intervention was more extensive in the ACT condition than in CBT. The difference between the two interventions was reasonably attributed to the intrinsic different goals of the therapy: while CBT is focused on the reduction of symptoms [24], providing an immediate—but not lasting—relief, ACT fosters psychological flexibility, considered the core mechanism of action of therapy [18].

In the field of health promotion, it was found that psychological flexibility fosters the adoption and the maintenance of behaviors which are driven by personal values and promotes an open, willing, and accepting attitude toward internal and external undesirable private events such as thoughts, emotions, and bodily sensations. Finally, it improves the ability to be present in the moment and face events in the context they happen [25].

Even if it has been well-established that psychological flexibility is the core mechanism of action in the ACT-based interventions, less is known about how each subcomponent acts.

To the authors’ knowledge, only one study [26] has previously explored the specific effect of two subcomponents of the ACT on treatment processes and outcomes in 15 adults seeking mental health treatment, founding that both the modules—one targeting acceptance and defusion, and one targeting values-based activation—produced improvements in psychiatric symptoms and quality of life, as well as improvements in specific therapeutic processes. However, no study has already assessed the specific effect of every single subcomponent of the psychological flexibility model, in weight management.

On the basis of these premises, the aim of the current study is to assess—within the context of a brief ACT-based intervention aimed to promote the adoption and maintenance of healthy lifestyle behaviors in a sample of Italian adult individuals with obesity—the specific effect of each subcomponent of the ACT model on the promotion and, most importantly, the maintenance of a healthy lifestyle.

The intervention will be part of a multidisciplinary 1-month rehabilitation program for weight loss.

## Methods

### Study design

An equivalent randomized controlled trial (RCT) with parallel groups will be conducted. We will perform a simple randomization with 1:1:1 allocation ratio using a computer-generated randomization (www.randomization.com). Allocation concealment will be ensured since all the patients will generate an anonymous code that will be associated to the randomization’s sequence generated by the program. Researchers will be blinded to the association made.

Since we already assessed the efficacy of an ACT-based intervention compared to a CBT-based intervention in the same context of obesity rehabilitation in a previous study [23], in this study, the aim was to compare the three subcomponents of the psychological flexibility model with each other.

Thus, participant enrolled for the study will be randomly allocated into three experimental conditions, targeting respectively each specific subcomponent of the psychological flexibility model:

1. Group *Engage*
2. Group *Openness*
3. Group *Awareness*

### Participants

Ninety Italian adult individuals with obesity (BMI ≥ 30) attending a multidisciplinary rehabilitation program for weight loss in a single clinical center in the North of Italy will be recruited for the study if they met the following criteria assessing in a clinical interview: (1) age between 18 and 65 years, (2) BMI ≥ 30, (3) being technology friendly to use wearable devices, and (4) written and informed consent to participate.

Exclusion criteria for the study are (1) any psychiatric disturbances (including eating disorders, diagnosed according to DSM 5 criteria) and (2) any medical conditions that could compromise participation at the study.

### Measures

Demographical and clinical data will be collected via a self-report form. Demographical data include gender, age, educational level, marital status, and work status.

Primary outcomes are physical (weight and BMI) and psychological conditions (PGWBI; OQ-45.2; DASS; DERS; DEBQ; AAQ-II) and adherence to a healthy lifestyle. Secondary outcomes are subcomponents of psychological flexibility (BVI, CAQ, I-CFQ, FFMQ). All the outcomes will be collected as follows.

#### Primary outcomes

##### Physical variables

Weight and height will be assessed to calculate body mass index (BMI = kg/m^2^).

##### Psychological conditions

Psychological well-being. The *Psychological General Well-Being Inventory* (PGWBI [27];). The Italian validation of Grossi and colleagues [28] consists of 22 self-administered items rated on a 6-point Likert scale, relative to six subscales that offer a measure of the level of subjective psychological well-being. Subscales are anxiety, depression, positive well-being, self-control, general health, and vitality. The internal consistency of the measure was good, with *α* values of subscales ranged from .61 to .85.

Treatment outcomes. The *Outcome Questionnaire-45.2* (OQ-45.2 [29];). The Italian version [30] is used to assess the effectiveness of clinical interventions. It is a self-report questionnaire composed of 45 items. Subscales are symptoms of distress, interpersonal relations, and social role functioning. The total *α* score obtained in the validated version on a clinical sample is excellent (*α* = .90).

Psychological inflexibility and experiential avoidance. The *Acceptance and Action Questionnaire* (AAQ-II [31];). The Italian version [32] is the most widely used self-reported questionnaire that measures psychological inflexibility and experiential avoidance. It is composed of 10 items, rated on a 7-point Likert scale; the internal consistency of the Italian validated version is good (*α* .83).

Depression, anxiety, and stress. The *Depression Anxiety and Stress Scale* (DASS-21 [33];). The Italian version [34] is administered as a measure of psychological distress. It is a self-report instrument that measures several negative internal states: depression, anxiety, and stress. It consists of 21 items rated on a 4-point Likert scale, ranging from 0 to 3. Internal consistency of the Italian validated version in the clinical sample was excellent with *α* value of total score of .92 and *α* values of subscales ranged from .83 to .91.

Emotional regulation. The Difficulties in Emotional Regulation Scale (DERS [35];). The Italian version [36, 37] is administered to assess difficulties in emotional regulation. This is a self-report questionnaire consisting of 36 items, rated on a 5-point Likert scale ranging from 1 (almost never) to 5 (almost always), which explores the following subscales: non-acceptance of negative emotions, inability to undertake purposeful behavior when experiencing negative emotions, difficulty in controlling impulsive behavior when experiencing negative emotions, limited access to emotion regulation strategies that are considered effective, lack of awareness of one’s emotions, and lack of understanding of the nature of one’s emotional responses. The internal consistency of the validated version on a non-clinical sample was good with *α* values of subscales ranged from .77 to .89. The *α* value of the total score was .92.

Emotional eating. The Emotional eating subscale of the Dutch Eating Behaviors Questionnaire [38]. The Italian version [39] is composed of 13 of 33 items of the DEBQ to assess emotional eating. Items are rated on a 5-point scale (1 = never, 5 = very often). Higher scores reflect higher levels of emotional eating. The internal consistency of the validated version of the emotional eating subscale in the overweight sample was excellent (*α* = .97).

#### Secondary outcomes

##### Subcomponent of the psychological flexibility model

Values. The *Brief Values Inventory* (BVI [40];). The Italian version [41] is composed of 12 items aimed to assess the success in living according to personal values. The internal consistency of the Success subscale is .70 in the Italian validation study.

Committed Action. The *Committed Action Questionnaire* (CAQ [42];). The Italian version [43] is an 8-item self-report questionnaire rated on a 7-point Likert scale, used to assess positive and negative aspects of the ability to engage themselves into committed actions driven by values. The internal consistency of the measure tested on a normative sample was good (*α* = .80).

Cognitive fusion. The *Italian-Cognitive Fusion Questionnaire* (I-CFQ [44];). The Italian version [45] is a 7-item questionnaire administered for the assessment of cognitive fusion. The internal consistency of the Italian version is excellent (*α* = .82).

Acceptance. The subscale “Nonjudge” of the *Five Facet Mindfulness Questionnaire*, described below, is used as a measure of acceptance. The internal consistency of the subscale in the validated version is good (*α* = .86).

Mindfulness. The Five Facet Mindfulness Questionnaire (FFMQ [46];). The Italian version [47] is a 39-item self-report questionnaire used as a measure of mindfulness. It is composed of five subscales: observe, describe, act with awareness, non-react, and nonjudge. The internal consistency of the total scale in the validated version is good (*α* = .86).

In particular, the BVI and the CAQ are administered to assess the Engagement subcomponent of the psychological flexibility model. The I-CFQ and the subscale *Nonjudge* of the FFMQ are administered to assess the Openness subcomponent of the psychological flexibility model. The FFMQ is administered to assess the Awareness subcomponent of the psychological flexibility model.

##### Lifestyle

Indicators of healthy lifestyle recorded using wearable devices are (1) daily minutes of moderate-to-vigorous physical activity, (2) daily total amount of calories, and (3) daily steps.

According to guidelines [48, 49], a healthy lifestyle requires a weekly total amount of moderate-to-vigorous physical activity of 150 min, daily energy intake around 80% of the basal energy expenditure estimated, and a weekly mean of 10,000 total daily steps.

### Procedures

Patients will be consecutively recruited for the study at the beginning of the rehabilitation program, after an interview conducted by clinical psychologists members of the research team, aimed to propose the research, assess the eligibility criteria, and ask an e-mail contact to send informed consent to participate.

The responsible of the study (GC) will obtain the informed consent from all participants of the study.

During the 1-month hospitalization, all participants will attend a multidisciplinary rehabilitation program composed by medical, nutritional physical, and psychological components. Patients will follow a hypocaloric balanced diet provided by dietitians. They will take part in a nutritional counseling program provided both in individual and group sessions focused on nutritional education, information on obesity and their health-related risks, setting personal goals for weight loss, weight management, behavioral changes, and prevention of relapses.

Patients will perform physical activity once a day (1 h) with trainers and physiotherapists consisting of programs of aerobic activity, postural gymnastics, and walking.

With respect to the psychological component, they will be randomly allocated into three experimental conditions targeting respectively one specific subcomponent of the psychological flexibility model:

1. Group *Engage*
2. Group *Openness*
3. Group *Awareness*

All conditions comprise two sessions, provided twice a week, lasting about 1 h each. Sessions are delivered by an expert clinical psychologist in ACT and will include experiential exercises and metaphors.

To assess the adherence to a healthy lifestyle following the rehabilitation program, outpatients are monitored for 1 month for diet and physical activity. At discharge, they receive wearable devices to monitor the adherence to a healthy lifestyle and are instructed on how to use wearable for the following 1 month.

At the beginning of the study (time 0), at the end of the intervention (time 1), and after 3 (time 2), 6 (time 3), and 9 months (time 4) from discharge, all demographical (such as gender, age, etc.), physical (weight and height to calculate BMI (kg/m^2^)), and clinical variables (PGWBI, OQ-45.2, DASS-21, DERS, DEBQ, BVI, CAQ, I-CFQ, FFMQ, AAQ-II) will be collected via an online survey (using Qualtrics platform).

In order to monitor the adherence of a healthy lifestyle after the period of rehabilitation, at discharge, participants will receive a wearable device for daily recording of habits regarding diet and physical activity. Monitoring will last 1 month after discharge.

We do not expect any adverse or unintended effects due to the trial participation. However, in case of any form of psychological discomfort, participants can consult the psychologist responsible for the division. Moreover, in case of any doubts or need for information, the participant can contact the responsible of the study. Once enrolled, patients may withdraw from the study at any stage and this will not affect their future treatment.

The clinical psychologist who will conduct the sessions, the participants, and the observers will be blinded to research aims.

All personal information about participants and records of all patients will be collected and preserved separately, in a secure place, into password-protected files kept for 5 years after the end of the trial.

All data collected will be stored in cloud computing and provided to researchers through a dedicated web platform.

The Medical Ethics Committee of Istituto Auxologico Italiano approved the study.

**Fig. 1:**
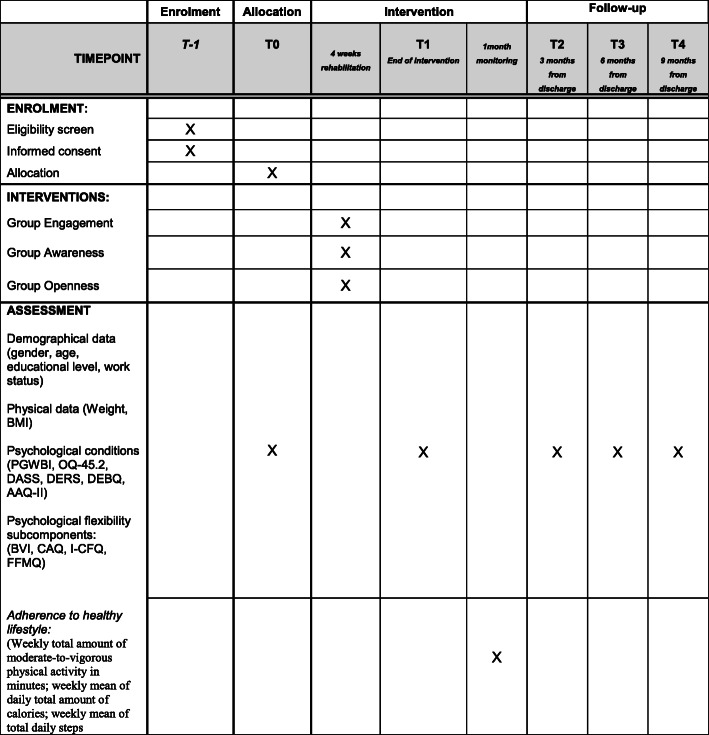
Schedule of enrolment, intervention, and assessment procedures

Schedule of enrolment, intervention, and assessment in the study is presented in Fig. 1.

### Intervention

#### Group Engagement

During this intervention, patients will have the opportunity to increase their motivation to change and encourage the engagement in committed actions, consistent with their life values. Patients are invited to reflect on what is important in their lives, which values make their life worth living, and which actions they could take to live a meaningful life, in accordance with personal values. The use of metaphors and experiential exercises will facilitate the process of exploring personal values, identifying life directions and related behaviors. For example, the 80th Birthday Party metaphor requires participants to imagine there is a party in honor of their birthday and the time comes when people are starting to give speeches and try to answer the question about what they want to hear people at the party say. This exercise helps patients in wondering what person they want to be with themselves and others.

#### Group Openness

Participants attending this intervention are guided to recognize and distance themselves from stressful thoughts, feelings, and sensations. They will learn to read suffering as part of human experience, without self-judgment and self-condemnation. Rather, the psychologist will encourage patients’ assumption of an open and acceptable approach to internal experiences. Throughout the session, the psychologist will help patients to reflect on their usual, but ineffective efforts to solve personal problems, and encourage the adoption of new responsive strategies based on acceptance and defusion from personal distress. An example of a metaphor used is The Passenger on a bus. In this metaphor, patients have to imagine to be a driver bus and his every thought is a passenger that gets on and off the bus. This exercise help patients to accept, defuse from, and reduce the power of their thoughts.

#### Group Awareness

The intervention comprises meditation exercises and experiences aimed to learn how to act intentionally with awareness about personal thoughts and sensations without automatically reacting. Participants are supported to recognize their actions and the context where they occur and learn to choose to respond with action consistent with their values and not automatically. The psychologist will propose breathing exercises, body scan, and other mindfulness experiences. Participants will be encouraged to sitting comfortably, close the eyes, feel themselves in contact with the present moment they are living, paying attention to their breath, noticing the rhythm, and any other aspect of the experience of breathing. Then, the therapist guides the participant’s attention on the body, noting any part of their body from the head to feet and then, the sounds around, any noises that could distract their attention on themselves.

### Treatment fidelity

The research group comprises licensed and clinical psychologists, researchers, and doctoral student experts in the field of clinical interventions in health care settings and research. Group sessions will be administered by a clinical psychologist with many years of expertise in ACT clinical practice both in individual and group settings, blinded to research scopes. The structure and the content of interventions are consistent with ACT theory. Two bachelor-level observers, also blinded to the research, after a period of training, will attend at least 20% of sessions to evaluate the adherence to intervention protocol, the coverage of contents, and the use of any additional strategies. They will use a checklist detailed for all the content of each intervention, including the process targeted, experiential exercises, and metaphors planned for each session. Coders have to achieve a minimum of 80% reliability with the expert trainers and each other. With lower level of agreement, the data will be dismissed [26].

### Statistical analysis

Data will be analyzed basing on intention-to-treat. This analysis includes all randomized patients in the groups to which they are randomly assigned, regardless of their adherence with the entry criteria, regardless of the treatment they actually received, and regardless of subsequent withdrawal from treatment or deviation from the protocol. Incidence, mechanisms, and patterns of missing data will be explored. To assess if missing data follow a Missing Completely At Random, Little’s MCAR test will be employed. Missing data lower than 5% will be considered negligible. In case of further amount of missing data, multiple imputation will be used.

Sample size has been calculated for a 3 (between) × 4 (within) ANOVA using G*Power software (release 3.1.3) [50]. Setting alpha to 0.05, power to 0.80, and possible dropout, a total sample of 90 is considered enough to detect a small effect size (*f* = 0.20).

Descriptive statistics (such as means, standard deviations, skewness, and kurtosis) will be conducted to explore the characteristics of the sample and assess for the normal distribution of the variables. Differences between baseline characteristics of the sample will be explored using one-way analysis of variance (ANOVA). To assess relations between variables, correlational analysis will be performed. Chi-squared for categorial variables and Pearson’s correlations for continuous variables will be used. To assess the effectiveness of the intervention, mixed between-within 3 (conditions) × 4 (times) repeated measure ANOVAs will be conducted to examine changes from time 0 to time 1, 2, 3, and 4 in means of weight, BMI, and means of scores PGWBI, OQ-45.2, DASS, DERS, DEBQ, AAQ-II, BVI, CAQ, I-CFQ, and FFMQ, between three groups. Corrected effect sizes (Hedges’s *g*) and significance at 95% confidence interval (95% CI) will be calculated for both between-group and within-group differences.

In order to assess adherence to healthy lifestyle recommendations, total means of each daily registered indicator of a healthy lifestyle will be calculated, as well as differences of the same parameters between groups, using one-way ANOVAs.

Analysis will be performed using IBM Statistical Package for the Social Sciences (SPSS) version 26.

## Discussion

Traditional CBT-based intervention for obesity management targets the adoption to a healthy lifestyle through the promotion of cognitive restructuring and skills training, such as coping, problem solving, stimulus control, and enhancing self-efficacy [6]. Even if such programs have been recognized effective in promoting the adoption of a healthy lifestyle, they are focused on treating symptoms and problems rather than promoting adaptive skills. Moreover, the maintenance of behavioral change remains challenging and alternative interventions are needed.

ACT seems a promising candidate to be considered an effective alternative to the gold standard for the treatment of obesity, but additional evidence of its effectiveness and a better understanding of its mechanism of action is required. For this reason, the current study protocol has been conceived.

The present study has several strengths. Firstly, to the best of the author’s knowledge, this is one of the first attempts to evaluate the specific impact of each subcomponent of psychological flexibility on promoting behavioral change and, most importantly, behavioral maintenance in a sample of Italian adult individuals with obesity who are seeking treatment for obesity management. Our research may provide additional evidence concerning the efficacy of ACT and may contribute to advance knowledge on how psychological flexibility’s subcomponent act in promoting behavioral change.

Then, our study involves both direct and indirect measures. While weight, BMI, and all the clinical variables are collected via a self-report form, a direct measure of healthy lifestyle habits will be collected using wearable devices for 1 month after discharge. Wearables are a novel device category that has become very popular in recent years [51]. They offer a direct measure of behaviors; thus, they do not suffer from bias of self-report measure.

Finally, two measures of follow-up (at 6 and 12 months after discharge) will allow to assess long-standing adoption of healthy lifestyle of participants.

Several limitations of the study should be considered. Firstly, the sample will be exclusively composed of hospitalized individuals, which may induce a selection bias. Thus, generalization to patients from different settings should be carefully done. Likewise, the high specific context in which the study will take place will make the result less generalizable.

Despite these limitations, this study will contribute to clarify the mechanism of action of the subcomponent of the psychological flexibility model and understand its impact on the promotion of a healthy lifestyle.

## Trial status

Protocol version: protocol version 1.1, date 19 February 2021

Data collection start: March 2021; data collection complete: March 2022.

## Data Availability

The datasets generated and/or analyzed during the current study will be available from the corresponding author on request. Records of all patients will be kept separately in a secure place. We plan to communicate trial results to participant with a final report provided via e-mail. Result will be available for the scientific communication and health care experts via publication on a scientific journal and participation into national and international congresses. We do not plan to deliver a completely deidentified dataset.

## Abbreviations

CBT: Cognitive behavioral therapy
ACT: Acceptance and commitment therapy
RCT: Randomized controlled trial
BMI: Body mass index
PGWBI: Psychological General Well-Being Inventory
OQ-45.2: Outcome Questionnaire-45.2
DASS: Depression Anxiety and Stress Scale
DERS: Difficulties in Emotional Regulation Scale
DEBQ: Dutch Eating Behaviors Questionnaire
BVI: Brief Values Inventory
CAQ: Committed Action Questionnaire
I-CFQ: Italian-Cognitive Fusion Questionnaire
AAQ-II: Acceptance and Action Questionnaire
MCAR: Missing Completely At Random
ANOVA: Analysis of variance
